# An elevated plus-maze in mixed reality for studying human anxiety-related behavior

**DOI:** 10.1186/s12915-017-0463-6

**Published:** 2017-12-21

**Authors:** Sarah V. Biedermann, Daniel G. Biedermann, Frederike Wenzlaff, Tim Kurjak, Sawis Nouri, Matthias K. Auer, Klaus Wiedemann, Peer Briken, Jan Haaker, Tina B. Lonsdorf, Johannes Fuss

**Affiliations:** 1 Human Behavior Laboratory, Institute for Sex Research and Forensic Psychiatry, Center of Psychosocial Medicine, University Medical Center Hamburg-Eppendorf, Martinistraße 52, 20246 Hamburg, Germany; 2 Institute of Systems Neuroscience, Center of Experimental Medicine, University Medical Center Hamburg-Eppendorf, Hamburg, Germany; 3 Department of Psychiatry and Psychotherapy, Center of Psychosocial Medicine, University Medical Center Hamburg-Eppendorf, Hamburg, Germany; 4 Medizinische Klinik und Poliklinik IV, Klinikum der Universität München, 80336 Munich, Germany

**Keywords:** Virtual reality, Approach, Avoidance, Risk assessment, Behavioral assay, Anxiety disorder, Acrophobia, Sensation seeking, Yohimbine, Lorazepam

## Abstract

**Background:**

A dearth of laboratory tests to study actual human approach-avoidance behavior has complicated translational research on anxiety. The elevated plus-maze (EPM) is the gold standard to assess approach-avoidance behavior in rodents.

**Methods:**

Here, we translated the EPM to humans using mixed reality through a combination of virtual and real-world elements. In two validation studies, we observed participants’ anxiety on a behavioral, physiological, and subjective level.

**Results:**

Participants reported higher anxiety on open arms, avoided open arms, and showed an activation of endogenous stress systems. Participants’ with high anxiety exhibited higher avoidance. Moreover, open arm avoidance was moderately predicted by participants’ acrophobia and sensation seeking, with opposing influences. In a randomized, double blind, placebo controlled experiment, GABAergic stimulation decreased avoidance of open arms while alpha-2-adrenergic antagonism increased avoidance.

**Conclusion:**

These findings demonstrate cross-species validity of open arm avoidance as a translational measure of anxiety. We thus introduce the first ecologically valid assay to track actual human approach-avoidance behavior under laboratory conditions.

**Electronic supplementary material:**

The online version of this article (doi:10.1186/s12915-017-0463-6) contains supplementary material, which is available to authorized users.

## Background

Avoidance of potential dangers at the cost of approach opportunities is a hallmark of anxious behaviors across all mammalian species [1]. Excessive and maladaptive avoidance contributes to the development and maintenance of anxiety disorders and prevents the extinction of fearful responding in humans [2–4] and rodents [5]. In animals, approach-avoidance conflicts are routinely assessed with behavioral assays that make use of rodents’ innate fear of heights (elevated plus-maze (EPM) [6, 7]), brightly illuminated (dark-light box [8]), or open (open field test [9]) spaces, which stand in conflict with curiosity and a drive for spontaneous exploration. Among these assays, the EPM is the most popular [6, 7], with over 8500 published papers so far (Web of Science, 2017). Creating an unconditioned approach-avoidance conflict, the EPM measures anxiety on a behavioral level by tracking adaptive behavior in the absence of explicit threat, punishment, or reward [6].

Although the EPM has been used for more than 30 years in laboratory rodents [6, 10] and other mammals [11–14] to test anxiety-related behaviors, a homologous test in humans is lacking. Instead, neurobiological research has relied on experimenter-designed proxies of human behavior such as eye or joystick movements [15]. Anxiety disorders are often inappropriate exaggerations of adaptive defensive behavior [6, 10, 16]. To understand mechanism of mental disorders, the study of such behavior is extremely valuable as it affects several diagnostic dimensions [16]. For example, the National Institute of Mental Health lists ‘behavior’ as one of the units of analysis in the Research Domain Criteria Matrix [17] although a behavioral measure for anxiety-related behavior has been lacking in humans. Thus, the Research Domain Criteria panel is currently empty [18].

Recently emerging technical and methodological advances enable behavioral tracking in humans (i.e., real-world body movements) and can be synergistically combined with novel mixed reality paradigms (a combination of virtual and real-world elements) providing controlled experimental conditions with high realism. This opens up new horizons in the development of standardized behavioral tests whose real-world implementation was, until now, too dangerous or laborious. Thus, well-established behavioral assays can be translated from rodents to humans, allowing the translational study of mechanisms of (anxiety) behaviors across species [19].

By translating the EPM to humans, we herein present a novel task in mixed reality that allows tracking of approach-avoidance behavior that is ecologically (i.e., resemblance with the real world) and ethologically (i.e., relevant for the species) valid. Based on comprehensive work in rodents, we hypothesize (1) that participants’ anxiety can be measured with identical behavioral outcome parameters as in rodents; (2) that avoidance of aversive open arms is related to acrophobia, while approach of open arms is related to sensation seeking in humans; and (3) that anxioselective drugs shift the balance between human approach and avoidance behavior as repeatedly shown in rodents [10].

## Results

### Human behavior on the EPM (Study 1)

**Fig. 1:**
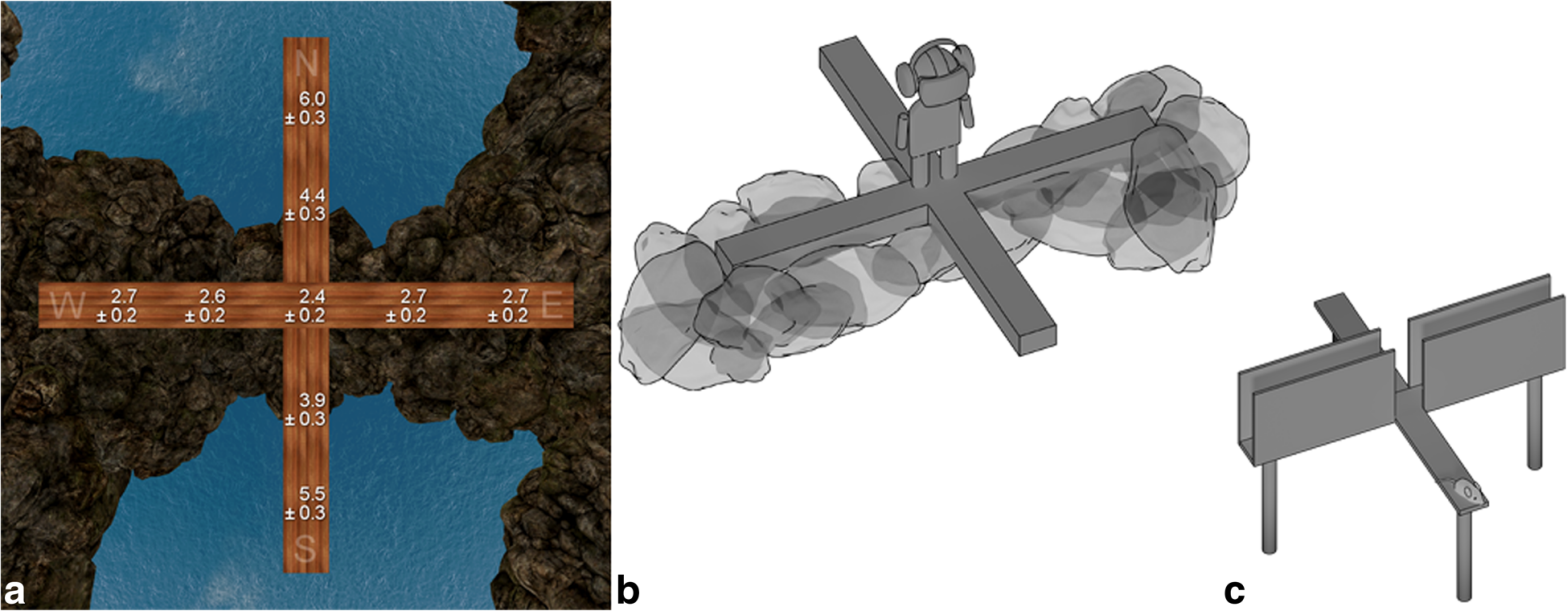
**a** Subjective anxiety ratings (*n* = 100; on a 0 *no anxiety* to 9 *very strong anxiety* scale) on different positions of the elevated plus-maze (EPM) in mean ± standard error. Open arms are in North (N) and South (S) direction, while closed arms are in East (E) and West (W) direction. Schematic depictions of the human (**b**) and rodent (**c**) EPM. Of note, the rocks surrounding the closed arms in the human EPM (**b**) only appear in the virtual environment, while the wooden real-world maze is placed on the floor of the laboratory. The closed arms in the rodent EPM (**c**) are surrounded by walls

In study 1, we were first interested if human behavior is comparable to rodent behavior on the EPM. Healthy participants (*n* = 100) were thus behaviorally tested. In line with studies in rodents [6], participants spent significantly more time on safe fractions of the maze (closed arms and center [20]) and thereby avoided spending time on the aversive open arms fraction (t_99_ = –11.89; *P* < 0.001, *r* = 0.77) and moved slower on open compared to closed arms (t_99_ = –8.55; *P* < 0.001, *r* = 0.65). Importantly, these behavioral differences were mirrored in subjective anxiety ratings (Fig. 1a; F_3.4,238.5_ = 52.67; *P* < 0.001; η^2^_partial_ = 0.426).

More precisely, open compared to closed arms or center induced higher levels of anxiety (each position *P* < 0.05). Similarly, higher anxiety was reported on distal (far from center) compared to proximal (closer to center) positions of open arms (each *P* < 0.001). There were no further significant differences between fractions of the maze. Descriptively, 98% of participants reported having been anxious on the maze, while only two participants rated zero anxiety on the maze. Other subjective ratings (e.g., panic, tension) can be found in Additional file 1. Moreover, participants reported a high presence in the virtual environment (iGroup Presence Questionnaire: spatial presence, 8.1 ± 0.6; involvement, 2.3 ± 0.8; experienced realism, 4.8 ± 0.7) and most participants reported only slight side effects (SSQ total score, 20.7 ± 2.8; nausea, 16.4 ± 2.3; oculomotor symptoms, 14.7 ± 2.2; disorientation, 26.1 ± 5.4).

Behavioral parameters on the EPM inform about approach and avoidance behavior as well as general activity in rodents [6, 10]. The most established parameters to assess anxiety reflect avoidance of open arms, such as total time spent on open arms (time on open arms) and number of entries to open arms. Other, less conventional, parameters are latency for the first entry of an open arm (latency 1^st^ visit) and time until subjects reach the end of an open arm (latency endexploration) [21, 22], which are common for other rodent approach-avoidance assays such as the dark-light box [23, 24]. All four parameters measure the approach-avoidance conflict concerning aversive open arms. High latencies, few visits and shorter time spent on aversive open arms (i.e., avoidance of open arms) are interpreted as elevated levels of anxiety in rodents. We hypothesized that these parameters are also suitable to examine anxiety in the human EPM. These parameters were thus calculated from the movement tracking data. Moreover, other parameters that were rather attributed to locomotor activity in rodents [20, 25], such as total distance covered, time on closed arms and center, entries to closed arms, average velocity on open and closed arms, as well as time in immobility, were collected.

**Fig. 2:**
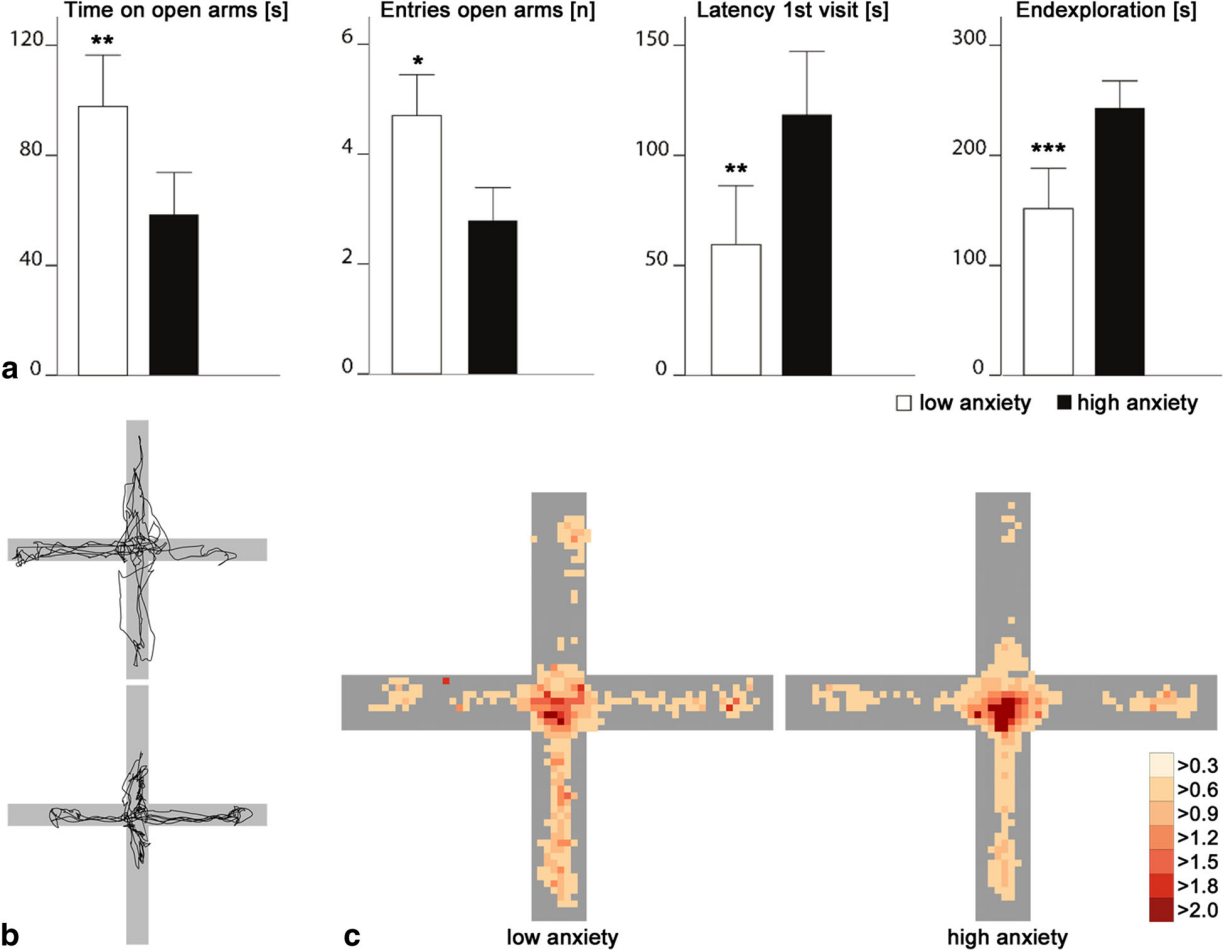
Barplots (**a**) represent mean values ± SEM for behavior on the elevated plus-maze (EPM) for low anxiety (LA) (*n* = 44; *white*) and high anxiety (HA) (*n* = 56; *black*) participants for the parameters (from *left* to *right*): time on open arms, entries of open arms, latency first visit, and endexploration of open arms + standard error. Exemplary trajectories for two participants are depicted under (**b**): The upper panel shows an LA participant and the lower panel an HA participant. Heatmaps (**c**) for LA (*left*) and HA (*right*) participants reveal where participants spent most time on the EPM. The average time spent at a position is shown in seconds – colors are defined in the legend (*right* bottom corner). For all depictions of the EPM, open arms are in North and South direction and closed arms in East and West direction. *P < 0.05, **P < 0.01, ***P < 0.001

The validity of these behaviors as measures of anxiety was established by pharmacological experiments and factor analyses in rodents [20, 25]. Here, we were able to ask participants directly after the test to rate their anxiety during EPM testing. We could thus compare behavioral parameters between participants with subjective high anxiety (HA) (*n* = 56; mean ± SE (min–max), 6.7 ± 0.2 (5–9)) and low anxiety (LA) (*n* = 44; mean ± SE (min–max), 2.5 ± 0.2 (0–4)) levels. Groups were defined using median split (median = 5, on a 0–9 scale). LA and HA differed significantly in their subjective anxiety on the maze in a *t* test (t_98_ = –17.87, *P* < 0.001, *r* = 0.87). In line with our hypotheses, we found that identical outcome parameters, as in rodents, were related to participants’ anxiety. LA spent significantly more time on open arms (t_98_ = 3.31, *P* = 0.001, *r* = 0.32; Fig. 2a), entered open arms more often (t_98_ = 4.05, *P* < 0.001, *r* = 0.38), and had a significantly shorter latency first visit (t_98_ = –2.94, *P* = 0.004, *r* = 0.28; Fig. 2a) and latency endexploration (t_98_ = –4.24, *P* < 0.001, *r* = 0.39; Fig. 2a).

Interestingly, other parameters that were rather attributed to locomotor activity in rodents, such as total distance covered, entries closed arms and time in immobility [25] were also significantly altered in LA (t_98_ = 4.09, *P* < 0.001, *r* = 0.38; t_98_ = 2.04, *P* = 0.044, *r* = 0.20; t_98_ = –3.84, *P* < 0.001, *r* = 0.36, respectively; Additional file 2). Thus, these parameters are not suitable to rule out locomotor effects of baseline locomotor activity on anxious behavior in humans as they do not reflect baseline locomotor activity. LA also moved faster on both open (t_98_ = 4.11, *P* < 0.001, *r* = 0.38) and closed (t_98_ = 3.20, *P* = 0.002, *r* = 0.31) arms. Table 1 displays a detailed description of behavioral data of LA and HA. Results of questionnaires for Study 1 can be found in Additional file 3.

**Table 1:** Behavioral measures of participants split for low anxiety (LA) and high anxiety (HA) and for all participants (*n* = 100) in Study 1

	LA (n = 44)				HA (n = 56)				All (n = 100)			
Measures	Mean	± SE	Min	Max	Mean	± SE	Min	Max	Mean	± SE	Min	Max
Latency open arm exploration (s)	59.6	13.3	3.2	300	118.4	14.3	65.6	300	92.6	10.3	3.2	300
Latency open arm end exploration (s)	151.6	18.4	6.4	300	242.9	12.5	30.6	300	202.7	0.1	6.4	300
Time on open arms (s)	97.9	9.3	0	213.3	58.4	7.7	0	233.7	75.8	6.2	0	233.7
Time on open arms (%)	32.6	3.1	0	71.1	19.5	2.6	0	77.9	25.3	2.1	0	77.9
Time on closed arms (s)	63.1	5.5	0	177.6	68.3	7.2	0	241.1	66.0	4.7	0	241.1
Time in center (s)	138.6	8.0	49.0	250.7	172.8	9.4	37.6	300	157.7	6.5	37.6	300
Time in center and on closed arms (s)	201.7	9.2	86.0	300	241.0	7.7	65.6	300	223.7	6.2	65.6	300
Time in immobility (s)	186.7	6.9	88.1	277.0	222.9	6.4	126.3	300	207.0	5.0	88.1	300
Average velocity on open arms (m/s)	0.051	0.004	0	0.100	0.032	0.003	0	0.110	0.041	0.002	0	0.098
Average velocity on closed arms (m/s)	0.069	0.004	0	0.130	0.050	0.004	0	0.119	0.058	0.003	0	0.129
Total distance covered (m)	14.8	0.8	4.5	29.5	10.6	0.6	0.7	20.1	12.4	0.5	0.7	29.5
Number of entries open arms (n)	4.7	0.4	0	7	2.8	0.3	0	7	3.6	0.3	0	10
Number of entries closed arms (n)	3.2	0.2	0	1	2.6	0.2	0	7	2.9	0.2	0	7

Measures are given as mean ± SE

*SE* standard error, *Max* maximum values, *Min* minimum

### Endocrinological and physiological responses to the EPM (Study 1)

**Fig. 3:**
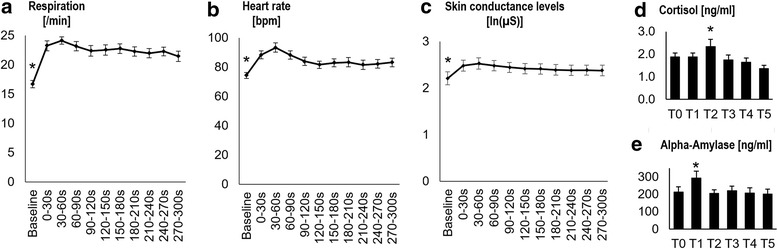
Physiological reactions to the elevated plus-maze (EPM) in Study 1. Barplots (**a**–**c**) show significant increases of heart (*n* = 30) and respiration rate (*n* = 29) as well as skin conductance levels (*n* = 11) throughout EPM testing compared to baseline. Barplots (**d** and **e**) show means for saliva cortisol (*n* = 28, upper panel) and alpha-amylase levels (*n* = 27, lower panel) before (T0) and directly after (T1) as well as at 15 min (T2), 30 min (T3), 45 min (T4), and 60 min (T5) after the experiment. Values are given as means ± standard error. *indicate significant differences between baseline and all other intervals for (**a**–**c**) and post hoc significance at *P* < 0.05 comparing cortisol at T2 to T1, T3, T4, and T5 and comparing amylase at T1 to all other time points

Earlier studies have shown that the EPM activates the rodent stress system and increases corticosterone levels [26]. We hypothesized that EPM testing also elicits a transient activation of the two endogenous human stress systems, namely the sympathetic nervous system and the hypothalamus–pituitary–adrenal axis. As expected, we found a main effect of time for salivary cortisol (F_2.2,60.6_ = 4.451, *P* = 0.013, η^2^_partial_ = 0.142) and alpha-amylase (F_5,130_ = 7.253, *P* < 0.001, η^2^_partial_ = 0.218; Fig. 3). In line with earlier experiments examining stress responses [27], the peak concentration of alpha-amylase was enhanced directly after EPM testing (significantly increased directly after compared to at all other time points; all *P* < 0.01), while cortisol peaked 15 min later (significantly higher at 15 min compared to directly after and at 30, 45, and 60 min (all *P* < 0.05), whereas before testing it was lower than at 15 min but on trend level (*P* = 0.09); Fig. 3).

Other markers for the immediate activation of the sympathetic nervous system are increased heart and respiration rate, as well as skin conductance levels (SCL). As expected, we found a significant effect of time on heart rate (F_3.4,97.2_ = 13.421, *P* < 0.001, η^2^_partial_ = 0.316), respiration rate (F_5.6,155.6_ = 16.102, *P* < 0.001, η^2^_partial_ = 0.365), and SCL (F_2,20.3_ = 9.961, *P* < 0.001, η^2^_partial_ = 0.499) when comparing the 11 time points assessed. All three parameters were significantly higher throughout the experiment compared to baseline (all *P* < 0.01 for heart rate, all *P* < 0.001 for respiration rate, and all *P* < 0.05 for SCL). Additional file 4 shows an exemplary depiction of the physiological reaction of one participant.

### Relating approach-avoidance to human traits (Study 1)

In rodents, open arm avoidance is related to elevated anxiety, while approach is related to novelty-seeking and curiosity [6]. We hypothesized that avoidance in humans is also related to acrophobic fear, whereas approach is associated with sensation seeking. Furthermore, measures of social anxiety and trait anxiety were included to test for the specificity of the findings against presumably unrelated forms of anxiety (i.e., social anxiety) or a general tendency for anxious temperament (i.e., trait anxiety). Hierarchical multiple regression analyses revealed, as expected, no significant contribution of anxious temperament (Spielberger State-Trait Anxiety Inventory; STAI) or social anxiety (Liebowitz Social Anxiety Scale; LSAS) on any of the behavioral parameters (Additional file 5). Variance in the main outcome measures was predicted by both acrophobia (Acrophobia Questionnaire; AQ), and sensation seeking (Sensation Seeking Scale Form V; SSSV), which, as expected, showed opposing directions (Additional file 6). In the final models, high acrophobia levels explained fewer entries of open arms (*R* ^2^ = 0.135, ß = 0.33, *P* = 0.001), less time on open arms (*R* ^2^ = 0.121, ß = 0.26, *P* = 0.01), and higher latency first visit (*R* ^2^ = 0.199, ß = –0.43, *P* < 0.001), while high sensation seeking levels explained longer time on open arms (*R* ^2^ = 0.121, ß = –0.21, *P* = 0.038) as well as shorter latency endexploration (*R* ^2^ = 0.189, ß = 0.23, *P* = 0.021). Interestingly, sensation seeking only influenced latency endexploration in participants with low acrophobia, but not with high acrophobia, being reflected in a significant SSSV*AQ interaction (*R* ^2^ = 0.189, ß = 0.23, *P* = 0.024).

### The influence of participants’ age and sex on anxiety-related behaviors (Study 1)

Anxiety symptoms [28] as well as 12-month and lifetime prevalence rates for all major anxiety disorders [29] are higher in women than in men differing across certain age groups [30]. In an explorative analysis, we therefore compared anxiety-related behaviors between men and women and correlated behavioral measures with participants’ age. Female participants exhibited more anxious behavior on the EPM concerning most outcome measures (Additional file 7). Moreover, latency open arm exploration (*R* = 0.223, *P* = 0.026), number of entries on open arms (*R* = –0.237, *P* = 0.018), and average velocity on open (*R* = –0.201, *P* = 0.45) and on closed arms (*R* = –0.250, *P* = 0.012) were found to be correlated with participants’ age.

### Demonstrating behavioral consequences of anxioselective drugs (Study 2)

**Fig. 4:**
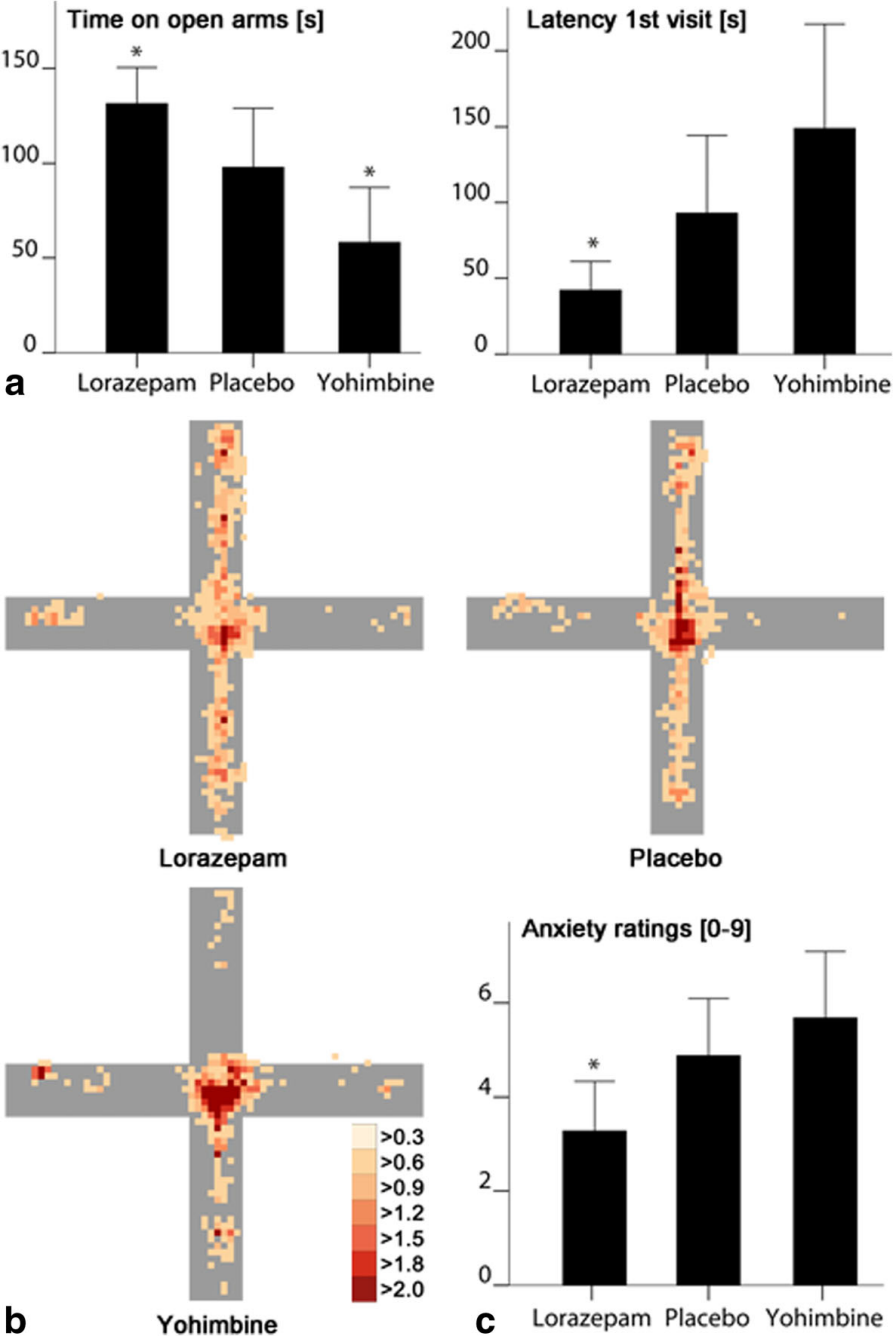
Barplots depicting behavior of participants (**a**) from lorazepam- (*n* = 18), placebo- (*n* = 17), and yohimbine-treated (*n* = 16) groups concerning time on open arms and latency first visit as well as subjective anxiety ratings (**c**). Heatmaps (**b**) for the groups reveal where participants spent most time on the elevated plus-maze. The legend shows the average time spent at a position in seconds. Open arms are in North and South direction and closed arms in East and West direction. Values are given as means ± standard error. *P < 0.05 significance in post hoc tests in comparison to the placebo group

In rodents, avoidance behavior has been shown to be sensitive to anxioselective compounds. We thus aimed to pharmacologically modulate behavior on the EPM using anxiolytic (lorazepam) and anxiogenic (yohimbine) substances in healthy participants in a placebo controlled, randomized, and double blind study (Study 2; *N* = 51). As hypothesized, pharmacological treatment significantly affected time on open arms (F_2,48_ = 7.529, *P* = 0.001, η^2^_partial_ = 0.239), with longer time in the lorazepam compared to the placebo group (t_33_ = 1.86, *P* = 0.036, *r* = 0.31; Fig. 4a), and a significantly shorter time in the yohimbine compared to placebo group (t_31_ = 1.86, *P* = 0.037, *r* = 0.32; Fig. 4a). Moreover, latency first visit significantly differed between groups (F_2,48_ = 4.720, *P* = 0.013, η^2^_partial_ = 0.164), with a shorter latency in the lorazepam compared to the placebo group (t_33_ = –1.90, *P* = 0.034, *r* = 0.31; Fig. 4a) and a descriptively higher latency in the yohimbine group compared to placebo only on trend level (t_31_ = –1.31, *r* = 0.22, *P* < 0.100). All other parameters did not show significant group differences. Table 2 shows a detailed overview of behavioral data.

**Table 2:** Behavioral measures for Study 2 comparing participants from lorazepam, placebo, and yohimbine groups

Measures	Lorazepam				Placebo				Yohimbine			
	Mean	± SE	Min	Max	Mean	± SE	Min	Max	Mean	± SE	Min	Max
Latency open arm exploration (s)	42.1	9.5	5.3	113.8	92.9	25.8	9.8	300	148.9	34.4	4.2	300
Latency open arm end exploration (s)	133.6	26.6	15.5	300	177.1	28.7	17.6	300	193.4	31.7	11.5	300
Time on open arms (s)	131.3	9.6	52.8	187.4	97.8	15.5	0	195.0	58.1	14.6	0	146.7
Time on open arms (%)	43.8	3.2	17.6	62.5	32.6	5.2	0	65.0	19.4	4.9	0	48.9
Time on closed arms (s)	46.1	5.6	10.8	105.3	39.1	9.5	0	165.3	53.3	15.4	0	218.2
Time in center (s)	122.1	9.7	77.0	207.8	162.4	18.4	79.4	300	187.9	19.5	81.1	300
Time in center and on closed arms (s)	168.2	9.5	111.9	246.4	201.6	15.6	104.2	300	241.2	15.6	152.6	300
Time in immobility (s)	198.2	8.4	131.6	269.5	205.7	15.7	86.0	300	219.4	13.2	114.6	290.8
Average velocity on open arms (m/s)	0.044	0.004	0.019	0.083	0.040	0.007	0	0.099	0.032	0.007	0	0.081
Average velocity on closed arms (m/s)	0.074	0.006	0.032	0.129	0.070	0.012	0	0.148	0.061	0.011	0	0.139
Total distance covered (m)	13.6	1.0	6.5	25.1	12.6	1.8	0	27.9	11.1	1.4	3.8	22.9
Number of entries open arms (n)	4.4	0.6	0	11	4.3	0.8	0	10	2.9	0.7	0	9
Number of entries closed arms (n)	2.9	0.3	1	6	2.4	0.4	0	5	2.1	0.4	0	5

Measures are given as mean ± SE

*SE* standard error, *Min* minimum, *Max* maximum values

The pharmacological challenge also exerted the expected effect on participants’ subjective anxiety ratings (F_2,48_ = 4.070, *P* = 0.023, η^2^_partial_ = 0.145; Fig. 4c), with significantly anxiolytic effects in the lorazepam compared to the placebo group (t_33_ = –2.00, *P* = 0.027, *r* = 0.34) and descriptively anxiogenic effects of yohimbine compared to placebo (t_31_ = 0.87, *P* = 0.196, *r* = 0.15). Additional descriptions of group comparisons regarding subjective ratings can be found in Additional file 8. The main outcomes were corroborated with an additional MANCOVA including age and sex as covariates (time on open arm F_2,46_ = 6.926, *P* = 0.002, η^2^_partial_ = 0.231; latency first visit F_2,46_ = 4.575, *P* = 0.015, η^2^_partial_ = 0.166; subjective ratings of anxiety F_2,46_ = 3.829, *P* = 0.029, η^2^_partial_ = 0.143).

Replicating the psychophysiological effect observed in Study 1, a significant effect of time on all three measures was revealed (heart rate F_3.8,148.8_ = 26.832, η^2^_partial_ = 0.408; respiration rate F_5.9,254.2_ = 26.612, η^2^_partial_ = 0.364; SCL F_3.1,103.2_ = 37.713, η^2^_partial_ = 0.533; all *P* < 0.001). Post hoc, all parameters at all time points showed significantly higher values compared to baseline (each *P* < 0.001). Moreover, there was a significant time*treatment interaction for heart rate (F_7.6,148.8_ = 2.242, *P* = 0.030, η^2^_partial_ = 0.103) but without significant post hoc effects.

## Discussion

Using a mixed reality paradigm, we herein demonstrate a translation of the EPM from rodents to humans. This is, to our knowledge, the first ecologically valid assay to track actual human approach-avoidance behavior in the laboratory to assess anxiety on a behavioral level.

Other approaches to translate behavioral assays into humans have only emerged recently. For example, the open field test [31] and Morris water maze [32] were successfully translated. However, both tasks require immense logistical efforts limiting comparability and standardization across laboratories. It would thus be advantageous to develop these tasks in virtual reality [19], as has been done with fear conditioning paradigms [33–35]. Other approaches used computer game paradigms for the radial arm [36] and Morris water maze [37] or approach-avoidance conflicts [38–41]. These computer games, though highly standardized, have the disadvantage of lower immersion compared to real-world, virtual- or mixed-reality paradigms and they only assess proxies of human behavior. Importantly, the human EPM is easily transferrable to other laboratories allowing comparability and grouping of data across laboratories. With the herein performed translation, further studies to identify species-conserved mechanisms likely implicated in anxiety and anxiety disorders are feasible. Translation of rodent tasks to humans could thus help to bridge preclinical science and clinical studies and may have an important role in the development of new anti-anxiety drugs [19].

Our data indicate good validity according to common definitions [42]. Firstly, presence and immersion in the mixed-reality were high. Moreover, participants’ anxiety could be observed by experimenters as participants often gasped at the beginning of the paradigm and moved precariously and slower on open arms (see, for example, the deep inhalation in Additional file 4 in response to EPM start). Thus, face validity (the ability of a test to measure what it is supposed to measure) can be assumed. Secondly, content validity (the extent to which a measure represents all facets of a given construct) was fulfilled – on a physiological level, the EPM stimulated the sympathetic nervous system and the hypothalamus–pituitary–adrenal axis, demonstrated by a rise in SCL, heart and respiration rate at the beginning of the experiment and elevated salivary alpha-amylase and cortisol levels after behavioral testing. On a behavioral level, participants spent most time on save compartments of the EPM (i.e., center and closed arms) and moved slower on open versus closed arms. On a subjective level, participants stated after the experiment that they had felt more anxious on open versus closed arms and center. Thirdly, concurrent validity (the measure in question is the same as another outcome assessed at the same time) can be assumed as we found a high correlation between subjective anxiety ratings and behavioral outcomes – HA participants displayed significantly more avoidance of aversive open arms compared to LA. Fourthly, predictive validity (the ability to predict behavior on a related measure) was met as we found significant associations of behavioral measures with trait measures of acrophobia and sensation seeking. Interestingly, acrophobia and sensation seeking both influenced behavior in opposite directions, which is in line with earlier theories that all emotional behaviors are controlled by at least two opponent motivational systems [43]. While acrophobia was related to avoidance (i.e., higher traits had longer latencies), sensation seeking was related to approach (i.e., shorter latencies). In rodents, it was demonstrated that anxioselective compounds, brain lesions, environmental factors, and genetic manipulations can shift the balance of the conflict between approach and avoidance [6, 43, 44]. To further prove predictive validity, we corroborated this bidirectional sensitivity of the EPM to manipulations of anxiety [7] by anxioselective effects of lorazepam and yohimbine in humans. Since construct validity (the ability of a test to measure the intended construct) is met if content and criterion (i.e., concurrent and predictive) validity are fulfilled, we can also assume construct validity. To our knowledge, this is the first validation of a human approach-avoidance test for innate, unconditioned anxiety that measures actual human behavior.

### Outlook and limitations

Our data were obtained from a healthy sample spanning a large dimension of anxiety-related traits. Future work might focus on individuals that are at the extreme ends of these dimensions. Moreover, to study treatment effects, repeated exposure to the EPM needs to be implemented in the future to perform longitudinal research, as in rodents [45]. The virtual part of the human EPM can be easily adapted (e.g., height of the maze, incentives for approach behavior, a different environment or inclusion of avatars as assisting partners) for longitudinal research and to suit populations with different needs such as children, elderly people, or psychiatric patients. Although we found in explorative analyses that age and sex influence anxiety-related behavior on the human EPM, these factors need to be addressed in the future with a larger sample size, including menstrual cycle phase and contraceptive use as co-variates, as well as more participants from older age ranges as only 15% of participants from our sample in Study 1 were aged between 30 and 40 years and only 4% between 40 and 50 years.

## Conclusion

Maladaptive or excessive avoidance is a hallmark of anxiety disorders and places a significant burden on affected patients. To date, research on anxiety disorders was complicated by the absence of a standardized laboratory test to study actual approach-avoidance behavior in humans. With the herein developed human EPM, anxiety can be inflicted and measured in an ambiguously threatening environment in mixed reality. Thus, we introduce a standardized and easily transferable paradigm for the research of human anxiety on a behavioral level. Identical outcome parameters in the human and rodent EPM facilitate translational research across species.

## Methods

### Participants and procedures

Healthy individuals aged between 18 and 50 years were invited to participate through a variety of means, including a popular local website for biomedical research recruitment, word of mouth, and a notice at local Universities. All laboratory procedures were performed between 1 to 6 pm for Study 1 and between 9 am to noon for Study 2. All participants received a telephone interview before inclusion to rule out somatic or psychiatric disorders as well as current drug use. Participants agreed to abstain from eating, drinking, smoking, and physical exercise for 2 hours prior to testing. Participants were informed about the procedure and gave written informed consent. All experiments were approved by the local ethics committee (Ärztekammer Hamburg, Germany) and the study was conducted in accordance with the good clinical practice guidelines as defined in the Declaration of Helsinki (2013).

For Study 1, 104 healthy participants were recruited. For pilot testing, a first subgroup of participants (*n* = 33; male = 16, female = 17) were recruited and psychophysiological recordings and saliva sample collection 5 min before (T0), directly after (T1), and 15 (T2), 30 (T3), 45 (T4), and 60 min (T5) after behavioral testing were performed only in this subgroup. The subjective experience of the virtual reality immersion was assessed using iGroup presence questionnaire [46] and side effects were measured using simulator sickness questionnaire [47]. Additionally, 71 participants were recruited as part of a larger ongoing data collection within the framework of a collaborative research center (SFB TRR 58). Participants provided personality- and anxiety-related questionnaires and were subjected to behavioral testing on the EPM. Participants were screened to be free from psychiatric disorders by the MINI diagnostic interview [48] prior to inclusion in the study. Three participants were excluded due to technical problems and one because of not following the study instruction leading to a sample size of *n* = 100 (female = 64, male = 36; age = 26.2 ± 0.5 years).

For Study 2, 57 participants (female = 34, male = 23; age = 26.9 ± 0.6 years) randomly received 1 mg of the benzodiazepine lorazepam, 20 mg of the alpha-2-adrenergic receptor antagonist yohimbine, or placebo in a double blind manner 60 min before behavioral testing. Six participants were excluded from behavioral testing or the analyses due to technical problems (*n* = 2) or strong side effects (‘strong sedation’, *n* = 4) before behavioral testing (Additional file 9). From the remaining 51 participants (female = 29, male = 22; age = 27.3 ± 0.6 years), 18 received lorazepam, 17 placebo, and 16 yohimbine. There were no significant differences concerning sociodemographic parameters and trait questionnaires between the three groups (Additional file 10). In all participants of Study 2, psychophysiological recordings were performed, while analyses of endocrinological measurements were not feasible because interaction of the medication with alpha-amylase levels was suspected and participants were tested in the morning, making an overshadowing of cortisol responses likely.

Four anxiety-related questionnaires from the collaborative research center sampling were chosen a priori; these were STAI [49], AQ [50], LSAS [51], and SSSV [52]. After behavioral testing, participants rated their anxiety level on the EPM on a scale from 0 (no anxiety) to 9 (very strong anxiety) as well as their anxiety level on different positions of the EPM. Moreover, we asked for side effects and other emotions (such as ‘having panic’, see Additional files 1 and 8) on a scale from 0 (not at all) to 9 (very strongly). For Study 2, state anxiety and side-effects of medication were assessed 5 min before behavioral testing.

### Human EPM

The human EPM consists of a real-world wooden maze combined with a representation of this maze in virtual reality. The real-world maze consists of four wooden arms (width 30 cm, height 20 cm). Each arm has a length of 175 cm, covering in total 350 × 350 cm, within an experimental room (550 × 550 cm) with two virtual reality tracking systems (HTC Vive Base Station®, Seattle, USA) attached at 250-cm height at opposite walls. Participants entered the room with closed eyes and were guided by one of the experimenters towards the maze. Participants received a headset (HTC Vive®, Seattle, USA) and noise canceling headphones (Bose QuietComfort 35®, Framingham, USA) and were instructed to open their eyes. After checking the vision of participants in a baseline graphical environment, the virtual reality software (A+ cross®, VirtualRealWorlds.com, Germany) was started. Participants found themselves in a 550 × 550 cm large virtual room with a virtual wooden plus-maze (350 × 350 cm) in front of them. Importantly, the virtual reality plus-maze and the physical real-world plus-maze had the same shape, material and size as well as position in the virtual and real world. A recorded voice instructed participants to step on the maze and walk slowly towards the center of the maze where participants had to wait for 60 s to allow for baseline measurements. Further, they were instructed that they would be allowed to explore the environment on the maze once the scene had changed. The behavioral experiment started after 90 s and participants found themselves in a new environment. In the new scenario, only the virtual plus-maze remained unchanged. Instead of being in a virtual room, the maze was placed on a virtual rocky mountain surrounded by water. Two opposite arms (here termed closed arms as in the rodent EPM) and the center of the maze were surrounded by rocks, while the other arms reached out over the water, which was roughly 55 m below (open arms). Simultaneously with the change of the virtual environment, two ventilators were started in the experimental room to increase presence in the virtual environment, as previously suggested [53, 54]. The ventilators were placed at the end of the arm that participants were initially facing in order to give the impression of a cool wind from ahead. Participants were allowed to explore the EPM for 300 s. After the scenario ended, participants removed their headsets and left the room. Additional file 11 shows simultaneous video recordings from different perspectives during the EPM.



**Additional file 11:** Movie 1. Simultaneous video recordings from different perspectives during EPM performance. Of note, two authors participated in video filming, thus no real participant is shown. (AVI 276316 kb)


### Data recording and synchronization of the EPM

Before experimental testing, the virtual maze was synchronized with the real-world maze using a controller (HTC Vive controller®, Seattle, USA), a purpose-designed mounting device, and a purpose-designed software (A+ cross®). During the alignment of the virtual environment and the physical maze, the calibration software (A+ cross®) axis-aligned the 3D world space coordinate system with the arms of the physical maze. The world space coordinate system was created with its origin at the center of the maze, its x-axis aligned with the closed arms, and its z-axis aligned with the open arms of the plus-maze.

Headset position and orientation during a running experiment were sampled at 5 Hz to obtain, for each sampling point $i$ at time $t$, a set of 3D positions ${\overrightarrow{p}}^i=\left({p}_x^i,{p}_y^i,{p}_z^i\right)$ and 3D orientations ${\overrightarrow{r}}^i=\left({r}_x^i,{r}_y^i,{r}_z^i\right)$. These measurements were subsequently analyzed automatically by the software (A+ cross®) to evaluate participants’ movement patterns on the maze. A sample point ${p}^i$ counts towards time spent on one arm if the absolute position on one of the axes, and thus distance from center, is larger than a pre-defined threshold value. The total time spent on one of the open arms (${t}_o$) and the closed arms (${t}_c$) is calculated as ${t}_{o,c}=\sum {t}^i\left[|{p}_{x,z}^i|> threshold\right]$.

The threshold value was determined manually by placing markers on the arms and evaluating the headset position for the time points when a participant stepped onto an arm with both feet. After this value had been obtained, it was used for the automatic analyses.

A participant’s change between the areas defined by the threshold value was evaluated at a maximum of one area change per second. This was done to prevent positional tracking jitter from creating false information about area changes for participants that were standing at a position very close to the threshold value.

In addition to the time spent in the different areas of the maze, all movements of the headset were calculated as average velocities ${\overrightarrow{v}}_{o,c}$ whenever ${p}_{x,z}^i> thres{h}_{\left[o,c\right]}$ and ${p}_{x,z}^{i-1}> thres{h}_{\left[o,c\right]}$.

Sample points for which the inverse case, ${p}_{x,z}^i> threshold$ and ${p}_{x,z}^{i-1}< threshold$, was measured were counted towards the total number of entries ${n}_{\left[o,c\right]}$ to one of the arms.

### Psychophysiological measurements

SCL, respiration, and heart rate (ECG) were recorded using BioNomadix wireless physiology devices and a BIOPAC MP150 data acquisition system and were analyzed using Acqknowledge 4.4.1 software (Biopac Systems, Goleta, CA, USA). Skin conductance Ag/AgCl electrodes were attached 10 min before behavioral testing to the index and middle fingers in Study 1 and on the palmar surface of the non-dominant hand in Study 2. Baseline levels were recorded 30 s before behavioral testing and average levels were compared to average levels of 30-s intervals during behavioral testing. In Study 1, 21 (66%) SCL datasets had to be excluded due to electrode detachment during behavioral testing. In Study 2, we changed positioning and type of electrodes and only 15 (29%) had to be excluded. Moreover, 3 (9%) respiration data from Study 1 and 5 (10%) from Study 2 as well as 2 (6%) ECG data from Study 1 and 9 (18%) ECG data from Study 2 had to be excluded due to poor data quality. To reduce exclusion rate, missing values due to movement artifacts were replaced with the means of nearby values if a value before and after the missing value existed.

### Endocrinological measurements

Participants received oral instructions on the correct use of the Salivette salivary collection device (Sarstedt AG, Nümbrecht, Germany). Samples were centrifuged and saliva stored at –80 °C until further analysis. Cortisol was determined by radioimmunoassay (DRG, Marburg, Germany). Inter- and intra-assay coefficients of variation were below 8% and detection limit was 0.5 ng/mL. Alpha-amylase was determined by using a commercial liquid phase enzymatic assay (RE80111, IBL International, Hamburg, Germany). Intra- and inter-assay coefficients of variance were below 7%, the detection limit was 25 U/mL.

### Statistical analyses

Statistical analyses were carried out using IBM SPSS Statistics 22.0 (IBM Corp., Armonk, NY, USA). A repeated measures ANOVA was performed to compare subjective anxiety ratings on different positions of the EPM (Fig. 1) as within-subject factors followed by post hoc comparisons using Bonferroni correction. Greenhouse–Geisser correction for sphericity was applied when appropriate. Paired *t* tests were used to compare behavior on open versus closed arms. For Study 1, participants were divided into subgroups for participants with subjective LA and HA ratings using median split. Two-sided *t* tests were used to compare behavioral measures between groups. According to our hypotheses, four hierarchical multiple regression analyses for time on open arms, latency first visit, latency endexploration, and entries open arm, including AQ as first, SSSV as second, and STAI as well as LSAS as the third step into the regression model, were performed. Thereafter, a second set of regression analyses was performed, including only the significant regressors as well as an interaction term of these significant regressors in a second step. To avoid multicollerality, mean centered values for factors were used.

Endocrinological measurements of Study 1 were analyzed using repeated measures ANOVA with time points (T0–T5) as the within-subject factor. In line with previous research [27], we hypothesized an increase of cortisol approximately 15 min after exposure to the EPM and of alpha-amylase directly after the experiment. Thus, one-sided post hoc paired *t* tests were performed comparing cortisol levels of T2 and alpha-amylase levels of T1 with all other time points. Psychophysiological data were analyzed using repeated measures ANOVA with the 11 intervals (baseline, 0–30 s, 30–60 s, etc.) as within-subject factors, followed by post hoc paired *t* tests comparing baseline with all other intervals. SCL data were transformed to logarithmic values (logarithmus naturalis (ln)).

In Study 2, sociodemographic factors and trait questionnaires were compared using MANOVA or χ^2^ tests of association between the three groups. Anxiety ratings and behavior were analyzed using MANOVA with treatment (lorazepam, yohimbine, placebo) as the factor. Earlier research provided strong evidence that the EPM is sensitive to pharmacological manipulation with benzodiazepines and yohimbine in rodents [10] and that 1 mg of lorazepam is anxiolytic and 20 mg of yohimbine is anxiogenic in humans [55, 56]. Thus, MANOVA was followed due to directed hypotheses by post hoc one-sided *t* tests to compare each pharmacological group with placebo. Psychophysiological data were analyzed in analogy to Study 1, but including treatment as a between-subject factor, followed by appropriate post hoc tests.

All data are given as mean ± standard error (SEM). Statistical significance was set at *P* < 0.05. Effect sizes are given as η^2^_partial_ for ANOVAs and as r for *t* tests. Original data used in this study are displayed in Additional files 12, 13, 14, 15 and 16.

## Additional files

Additional file 1: Table S1. Subjective ratings on a scale from 0 (not at all) to 9 (very strongly) that were collected after behavioral testing in Study 1 for all participants, as well as for subgroups with low and high subjective anxiety (LA, HA). (DOCX 16 kb)

Additional file 2: Figure S1. Total distance covered and entries of closed arms in Study 1 (a) and Study 2 (b). These measures are used to assess baseline locomotor activity in the rodent EPM. However, in the human EPM they also differ between LA and HA and are thus not suitable as measures for baseline locomotor activity. Some participants with very high anxiety did not move on the EPM due to behavioral inhibition or freezing. Thus, total distance covered can be affected by anxiety on the human EPM. (TIFF 93 kb)

Additional file 3: Table S2. Results of questionnaire data from Spielberger State-Trait Anxiety Inventory (STAI) trait subscore, Acrophobia Questionnaire (AQ), Liebowitz Social Anxiety Scale (LSAS), and Sensation Seeking Scale Form V (SSSV), including respective subscores from Study 1, given in mean ± standard error (SE), minimum (min), and maximum (max) values. (DOCX 20 kb)

Additional file 4: Figure S2. An exemplary depiction of the psychophysiological reaction to the elevated plus-maze of one participant. The black arrow marks the change from baseline testing (virtual room) to the beginning of the elevated plus-maze test (being in a new virtual environment on a rocky mountain above the sea) and the red arrow marks the moment when the participant entered the open arm for the first time. (TIF 1453 kb)

Additional file 5: Table S3. Results of regression analyses with Acrophobia Questionnaire (AQ), Sensation Seeking Scale Form V (SSSV), Spielberger State-Trait Anxiety Inventory (STAI), and Liebowitz Social Anxiety Scale (LSAS) (*P < 0.05, **P < 0.01, and ***P < 0.001). (DOCX 22 kb)

Additional file 6: Table S4. Results of multiple regression analyses with mean centered values for Sensation Seeking Scale Form V (SSSV) and acrophobia questionnaire (AQ) as well as interaction term AQ × SSSV; (*P < 0.05, **P < 0.01, and ***P < 0.001). (DOCX 20 kb)

Additional file 7: Table S5. Behavioral data of participants split for sex (N = 100). Mean ± standard error (SE), minimum (min), maximum (max) values. (DOCX 16 kb)

Additional file 8: Table S6. Subjective ratings on a scale from 0 (not at all) to 9 (very strongly) that were collected after behavioral testing in Study 2. (DOCX 16 kb)

Additional file 9: Table S7. Side effects after medication in Study 2. Data were collected directly before behavioral testing (i.e., 55 min after medication). (DOCX 19 kb)

Additional file 10: Table S8. Results of questionnaire data from Study 2 Spielberger State-Trait Anxiety Inventory (STAI) trait subscore, acrophobia questionnaire (AQ), Liebowitz Social Anxiety Scale (LSAS), and Sensation Seeking Scale Form V (SSSV) including respective subscores, given in mean ± standard error. (DOCX 17 kb)

Additional file 12: Supplemental file 1. Original data from Study 1. (CSV 23 kb)

Additional file 13: Supplemental file 2. Original endocrinological data from Study 1. (CSV 1 kb)

Additional file 14: Supplemental file 3. Original psychophysiological data from Study 1. (CSV 5 kb)

Additional file 15: Supplemental file 4. Original data from Study 2. (CSV 9 kb)

Additional file 16: Supplemental file 5. Original psychophysiological data from Study 2. (CSV 11 kb)
