# Supplementary material for: An elevated plus-maze in mixed reality for studying human anxiety-related behavior
Source: BMC Biol. 2017 Dec 21;15:125. doi: 10.1186/s12915-017-0463-6 (PMC5740602; doi:10.1186/s12915-017-0463-6)
Supplement: Supplementary file 2 — Subjective ratings on a scale from 0 (not at all) to 9 (very strongly) that were collected after behavioral testing in Study 1 for all participants, as well as for subgroups with low and high subjective anxiety (LA, HA). (DOCX 16 kb) [file 12915_2017_463_MOESM1_ESM.docx]

***Table S1.*** Subjective ratings on a scale from 0 *no at all* to 9 *very strongly* that were collected after behavioral testing in study 1 for all participants, as well as for subgroups with low and high subjective anxiety (LA, HA).

| *Measures* | ***All (n = 100)*** | | ***LA (n = 44)*** | | ***HA (n = 56)*** | |
| --- | --- | --- | --- | --- | --- | --- |
|  | **Mean** | **SEM** | **Mean** | **SEM** | **Mean** | **SEM** |
| I immersed into the scene | 7.8 | 0.1 | 7.2 | 0.2 | 8.3 | 0.1 |
| I had anxiety | 4.9 | 0.2 | 2.5 | 0.2 | 6.7 | 0.2 |
| It was threatening | 5.4 | 0.2 | 3.5 | 0.2 | 6.9 | 0.2 |
| I moved precariously | 6.7 | 0.2 | 5.0 | 0.3 | 8.0 | 0.1 |
| I had panic | 2.1 | 0.2 | 0.9 | 0.2 | 3.0 | 0.3 |
| I felt inner tension | 5.0 | 0.2 | 3.0 | 0.3 | 6.6 | 0.2 |
| I have inner tension now | 2.0 | 0.2 | 1.3 | 0.2 | 2.5 | 0.3 |
| I had somatic symptoms | 5.2 | 0.2 | 3.7 | 0.3 | 6.4 | 0.3 |
| I felt dizzy | 1.6 | 0.2 | 0.7 | 0.2 | 2.3 | 0.3 |
| I was paralyzed | 2.7 | 0.3 | 1.2 | 0.2 | 3.9 | 0.3 |
| I felt like being watched | 2.7 | 0.3 | 2.4 | 0.4 | 2.9 | 0.4 |
| I felt shame | 0.8 | 0.2 | 0.5 | 0.2 | 1.1 | 0.3 |
