# Supplementary figures and images for: An elevated plus-maze in mixed reality for studying human anxiety-related behavior
Source: BMC Biol. 2017 Dec 21;15:125. doi: 10.1186/s12915-017-0463-6 (PMC5740602; doi:10.1186/s12915-017-0463-6)

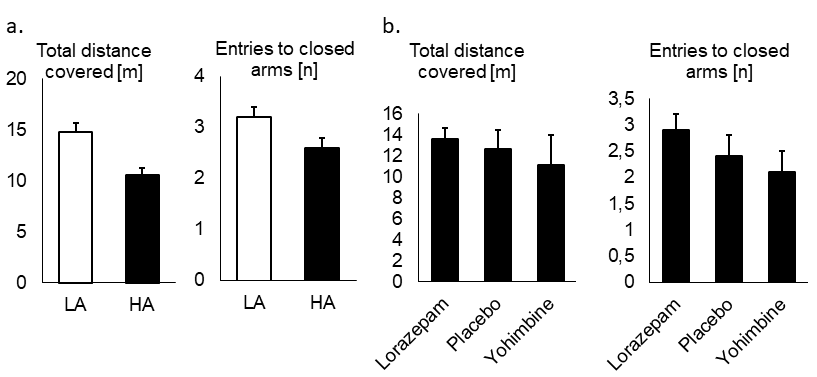

Supplement: Supplementary file 3 — Total distance covered and entries of closed arms in Study 1 (a) and Study 2 (b). These measures are used to assess baseline locomotor activity in the rodent EPM. However, in the human EPM they also differ between LA and HA and are thus not suitable as measures for baseline locomotor activity. Some participants with very high anxiety did not move on the EPM due to behavioral inhibition or freezing. Thus, total distance covered can be affected by anxiety on the human EPM. (TIFF 93 kb) [file 12915_2017_463_MOESM2_ESM.tiff]

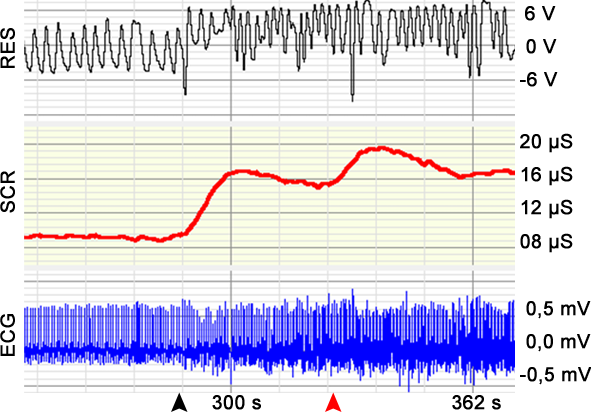

Supplement: Supplementary file 5 — An exemplary depiction of the psychophysiological reaction to the elevated plus-maze of one participant. The black arrow marks the change from baseline testing (virtual room) to the beginning of the elevated plus-maze test (being in a new virtual environment on a rocky mountain above the sea) and the red arrow marks the moment when the participant entered the open arm for the first time. (TIF 1453 kb) [file 12915_2017_463_MOESM4_ESM.tif]
