# Supplementary material for: An elevated plus-maze in mixed reality for studying human anxiety-related behavior
Source: BMC Biol. 2017 Dec 21;15:125. doi: 10.1186/s12915-017-0463-6 (PMC5740602; doi:10.1186/s12915-017-0463-6)
Supplement: Supplementary file 4 — Results of questionnaire data from Spielberger State-Trait Anxiety Inventory (STAI) trait subscore, Acrophobia Questionnaire (AQ), Liebowitz Social Anxiety Scale (LSAS), and Sensation Seeking Scale Form V (SSSV), including respective subscores from Study 1, given in mean ± standard error (SE), minimum (min), and maximum (max) values. (DOCX 20 kb) [file 12915_2017_463_MOESM3_ESM.docx]

***Table S2.*** Results of questionnaire data from Spielberger’s state-trait-anxiety inventory trait subscore (STAI), acrophobia questionnaire (AQ), Liebowitz social anxiety scale (LSAS), Sensation Seeking Scale Form V (SSSV) including respective subscores from study 1, given in Mean ± SE: standard error, Min: minimum, Max: maximum values.

|  | *All Participants* | | | | | *Low Anxiety* | | | | *High Anxiety* | | | |
| --- | --- | --- | --- | --- | --- | --- | --- | --- | --- | --- | --- | --- | --- |
|  | **N** | **Mean** | **± SE** | **Min** | **Max** | **Mean** | **± SE** | **Min** | **Max** | **Mean** | **± SE** | **Min** | **Max** |
| STAI trait | 100 | 35.6 | 0.8 | 21 | 59 | 35.1 | 1.2 | 21 | 51 | 36.0 | 1.0 | 23 | 59 |
| AQ - avoidance | 99 | 2.4 | 0.2 | 0 | 8 | 1.6 | 0.3 | 0 | 7 | 3.1 | 0.3 | 0 | 8 |
| AQ - anxiety | 99 | 15.7 | 1.4 | 0 | 63 | 10.7 | 1.8 | 0 | 63 | 19.5 | 1.8 | 0 | 59 |
| AQ - total | 99 | 18.2 | 1.5 | 0 | 64 | 12.4 | 1.9 | 0 | 64 | 22.6 | 2.0 | 0 | 64 |
| SSSV - thrill and adventure seeking | 97 | 6.3 | 0.3 | 1 | 10 | 6.7 | 0.4 | 1 | 10 | 5.9 | 0.3 | 1 | 10 |
| SSSV - disinhibition | 97 | 5.4 | 0.2 | 1 | 10 | 5.7 | 0.3 | 1 | 9 | 5.2 | 0.3 | 1 | 10 |
| SSSV - experience seeking | 97 | 6.5 | 0.2 | 2 | 10 | 6.5 | 0.3 | 2 | 10 | 6.6 | 0.2 | 3 | 10 |
| SSSV - boredom susceptibility | 97 | 4.0 | 0.2 | 1 | 10 | 3.9 | 0.3 | 0 | 10 | 4.1 | 0.2 | 0 | 7 |
| SSSV - total | 97 | 22.2 | 0.6 | 7 | 34 | 22.8 | 1.0 | 7 | 34 | 21.8 | 0.7 | 12 | 32 |
| LSAS - anxiety | 100 | 12.3 | 0.8 | 0 | 34 | 10.9 | 1.2 | 0 | 31 | 13.4 | 1.2 | 2 | 34 |
| LSAS - avoidance | 100 | 11.9 | 0.8 | 0 | 31 | 11.2 | 1.3 | 0 | 31 | 12.5 | 1.0 | 0 | 30 |
| LSAS - total | 100 | 24.2 | 1.5 | 2 | 60 | 22.1 | 2.3 | 2 | 60 | 25.9 | 2.0 | 2 | 60 |
