# Supplementary material for: An elevated plus-maze in mixed reality for studying human anxiety-related behavior
Source: BMC Biol. 2017 Dec 21;15:125. doi: 10.1186/s12915-017-0463-6 (PMC5740602; doi:10.1186/s12915-017-0463-6)
Supplement: Supplementary file 6 — Results of regression analyses with Acrophobia Questionnaire (AQ), Sensation Seeking Scale Form V (SSSV), Spielberger State-Trait Anxiety Inventory (STAI), and Liebowitz Social Anxiety Scale (LSAS) (*P < 0.05, **P < 0.01, and ***P < 0.001). (DOCX 22 kb) [file 12915_2017_463_MOESM5_ESM.docx]

***Table S3.*** Results of regression analyses with AQ, SSSV, STAI and LSAS; (*p < .05, **p < .01, and ***p < .001).

| Time on open arms | B | SE B | ß | T | p |
| --- | --- | --- | --- | --- | --- |
| Step 1 |  |  |  |  |  |
| Constant | 99.27 | 9.94 |  | 9.98 | <.001 |
| AQ | -1.20 | 0.43 | -.28** | -2.82 | .006 |
| Step 2 |  |  |  |  |  |
| Constant | 46.21 | 27.06 |  | 1.71 | .091 |
| AQ | -1.11 | 0.43 | -.26* | -2.64 | .010 |
| SSSV | 2.31 | 1.10 | .21* | 2.10 | .038 |
| Step 3 |  |  |  |  |  |
| Constant | 48,95 | 47.81 |  | 1.02 | .309 |
| AQ | -1.18 | 0.44 | -.28 | -2.70 | .008 |
| SSSV | 2.43 | 1.17 | .22 | 2.07 | .041 |
| LSAS | .56 | 0.52 | .13 | 1.09 | .278 |
| STAI | -.50 | 1.05 | -.06 | -0.48 | .635 |

Note: Step 1: R^2^ = .079** (p = 0.006), Step 2: ΔR^2^ = .042* (p = 0.038), Step 3: ΔR^2^ = .012 (p = 0.549)

| Latency 1^st^ visit | B | SE B | ß | T | p |
| --- | --- | --- | --- | --- | --- |
| Step 1 |  |  |  |  |  |
| Constant | 35.29 | 15.02 |  | 2.35 | 0.021 |
| AQ | 3.03 | 0.64 | .44*** | 4.71 | <0.001 |
| Step 2 |  |  |  |  |  |
| Constant | 69.61 | 41.66 |  | 1.67 | 0.098 |
| AQ | 2.97 | 0.65 | .43 | 4.59 | <0.001 |
| SSSV | -1.49 | 1.69 | -.08 | -0.88 | 0.379 |
| Step 3 |  |  |  |  |  |
| Constant | -16.49 | 72.84 |  | -.23 | 0.821 |
| AQ | 2.88 | 0.67 | .42 | 4.30 | <0.001 |
| SSSV | -0.88 | 1.79 | -.05 | -0.05 | 0.625 |
| LSAS | -1.01 | 0.79 | -.15 | -0.15 | 0.203 |
| STAI | 2.77 | 1.60 | .21 | 1.73 | 0.088 |

Note: Step 1: R^2^ = .192*** (p < 0.001), Step 2: ΔR^2^ = .007 (p = 0.379), Step 3: ΔR^2^ = .027 (p = 0.210)

| Latency endexploration | B | SE B | ß | T | p |
| --- | --- | --- | --- | --- | --- |
| Step 1 |  |  |  |  |  |
| Constant | 165.68 | 18.42 |  | 8.99 | <0.001 |
| AQ | 2.02 | 0.79 | .26* | 2.56 | 0.012 |
| Step 2 |  |  |  |  |  |
| Constant | 296.66 | 49.18 |  | 6.033 | <0.001 |
| AQ | 1.80 | 0.76 | .23* | 2.36 | 0.021 |
| SSSV | -5.69 | 1.99 | -.28** | -2.86 | 0.005 |
| Step 3 |  |  |  |  |  |
| Constant | 318.69 | 86.70 |  | 3.68 | <0.001 |
| AQ | 2.01 | 0.80 | .26 | 2.53 | 0.013 |
| SSSV | -6.20 | 2.13 | -.30 | -2.92 | 0.004 |
| LSAS | -1.09 | 0.934 | -.14 | -1.17 | 0.245 |
| STAI | 0.34 | 1.91 | .02 | 0.18 | 0.867 |

Note: Step 1: R^2^ = .066* (p = 0.012), Step 2: ΔR^2^ = .076** (p = 0.005), Step 3: ΔR^2^ = .015 (p = 0.445)

| Entries open arms | B | SE B | ß | T | p |
| --- | --- | --- | --- | --- | --- |
| Step 1 |  |  |  |  |  |
| Constant | 4.76 | 0.39 |  | 12.10 | <0.001 |
| AQ | -0.06 | 0.02 | -.35** | -3.54 | 0.001 |
| Step 2 |  |  |  |  |  |
| Constant | 3.43 | 1.09 |  | 3.16 | 0.002 |
| AQ | -0.06 | 0.02 | -.33 | -3.40 | 0.001 |
| SSSV | 0.06 | 0.04 | .13 | 1.32 | 0.192 |
| Step 3 |  |  |  |  |  |
| Constant | 4.09 | 1.91 |  | 2.13 | 0.036 |
| AQ | -0.06 | 0.02 | -.34 | -3.36 | 0.001 |
| SSSV | 0.06 | 0.05 | .13 | 1.23 | 0.223 |
| LSAS | 0.03 | 0.02 | .15 | 1.20 | 0.234 |
| STAI | -0.03 | 0.04 | -.10 | -0.82 | 0.416 |

Note: Step 1: R^2^ = .119*** (p < .001), Step 2: ΔR^2^ = .016 (p = 0.192), Step 3: ΔR^2^ = .014 (p = 0.480)
