# Supplementary material for: An elevated plus-maze in mixed reality for studying human anxiety-related behavior
Source: BMC Biol. 2017 Dec 21;15:125. doi: 10.1186/s12915-017-0463-6 (PMC5740602; doi:10.1186/s12915-017-0463-6)
Supplement: Supplementary file 7 — Results of multiple regression analyses with mean centered values for Sensation Seeking Scale Form V (SSSV) and acrophobia questionnaire (AQ) as well as interaction term AQ × SSSV; (*P < 0.05, **P < 0.01, and ***P < 0.001). (DOCX 20 kb) [file 12915_2017_463_MOESM6_ESM.docx]

***Table S4****.* Results of multiple regression analyses with mean centered values for SSSV and AQ as well as interaction term AQ x SSSV; (*p < .05, **p < .01, and ***p < .001).

| Time on open arms | B | SE B | ß | T | p |
| --- | --- | --- | --- | --- | --- |
| Step 1 |  |  |  |  |  |
| Constant | 77.29 | 6.12 |  | 12.62 | <.001 |
| AQ | 1.11 | 0.42 | .26* | 2.64 | 0.010 |
| SSSV | -2.31 | 1.10 | -.21* | -2.10 | 0.038 |
| Step 2 |  |  |  |  |  |
| Constant | 76.75 | 6.16 |  | 12.45 | <.001 |
| AQ | 1.03 | 0.43 | .24 | 2.37 | 0.020 |
| SSSV | -2.09 | 1.13 | -.19 | -1.86 | 0.067 |
| AQ*SSSV | -0.07 | 0.08 | -.09 | -0.87 | 0.386 |

Note: R^2^ = .121** for Step 1 (p = 0.003), ΔR^2^ = .007 (p = 0.386)

| Latency 1^st^ visit | B | SE B | ß | T | p |
| --- | --- | --- | --- | --- | --- |
| Step 1 |  |  |  |  |  |
| Constant | 90.39 | 9.43 |  | 9.56 | <.001 |
| AQ | -2.97 | 0.65 | -.43** | -4.59 | <.001 |
| SSSV | 1.49 | 1.69 | .08 | 0.88 | .379 |
| Step 2 |  |  |  |  |  |
| Constant | 91.42 | 9.47 |  | 9.65 | <.001 |
| AQ | -2.81 | 0.66 | -.41 | -4.23 | <.001 |
| SSSV | 1.09 | 1.73 | .06 | 0.63 | .532 |
| AQ*SSSV | 0.13 | 0.12 | .11 | 1.06 | .29 |

Note: R^2^ = .199*** for Step 1 (p < 0.001), ΔR^2^ = .010 (p = 0.290)

| Latency endexploration | B | SE B | ß | T | p |
| --- | --- | --- | --- | --- | --- |
| Step 1 |  |  |  |  |  |
| Constant | 202.82 | 11.13 |  | 18.22 | <.001 |
| AQ | -1.80 | 0.76 | -.23* | -2.36 | .021 |
| SSSV | 5.69 | 1.99 | .28* | .28 | .005 |
| Step 2 |  |  |  |  |  |
| Constant | 205.38 | 10.94 |  | 18.78 | <.001 |
| AQ | -1.40 | 0.77 | -.18 | -1.82 | .072 |
| SSSV | 4.68 | 2.00 | .23* | 2.34 | .021 |
| AQ*SSSV | 0.32 | 0.14 | .23* | 2.30 | .024 |

Note: R^2^ = .142** for Step 1 (p = 0.001), ΔR^2^ = .047* (p = 0.024)

| Entries open arm | B | SE B | ß | T | p |
| --- | --- | --- | --- | --- | --- |
| Step 1 |  |  |  |  |  |
| Constant | 3.67 | .25 |  | 14.95 | <.001 |
| AQ | 0.06 | .02 | .33* | 3.40 | .001 |
| SSSV | -0.06 | .04 | -.13 | -1.32 | .192 |
| Step 2 |  |  |  |  |  |
| Constant | 3.66 | .25 |  | 3.17 | <.001 |
| AQ | 0.06 | .02 | .32 | 0.02 | .002 |
| SSSV | -0.05 | .05 | -.12 | -.14 | .236 |
| AQ*SSSV | -0.00 | .00 | -.04 | -.01 | .706 |

Note: R^2^ = .135** for Step 1 (p = 0.001), ΔR^2^ = .001 (p = 0.706)
