# Supplementary material for: An elevated plus-maze in mixed reality for studying human anxiety-related behavior
Source: BMC Biol. 2017 Dec 21;15:125. doi: 10.1186/s12915-017-0463-6 (PMC5740602; doi:10.1186/s12915-017-0463-6)
Supplement: Supplementary file 8 — Behavioral data of participants split for sex (N = 100). Mean ± standard error (SE), minimum (min), maximum (max) values. (DOCX 16 kb) [file 12915_2017_463_MOESM7_ESM.docx]

**Table S5**: Behavioral data of participants split for gender (N = 100). Mean ± SE: standard error, Min: minimum, Max: maximum values.

|  | Female (n = 64) | | | | Male (n = 36) | | | |
| --- | --- | --- | --- | --- | --- | --- | --- | --- |
|  | Mean | ± SE | Min | Max | Mean | ± SE | Min | Max |
| Latency open arm exploration (s) | 121.3 | 13.7 | 3.8 | 300 | 41.5 | 11.0 | 3.2 | 260.4 |
| Latency open arm end exploration (s) | 242.3 | 12.0 | 18.0 | 300 | 132.3 | 19.3 | 6.4 | 300 |
| Time on open arms (s) | 51.6 | 5.9 | 0.0 | 174.0 | 118.7 | 10.4 | 4.0 | 233.7 |
| Time on closed arms (s) | 71.4 | 6.6 | 0.0 | 241.1 | 56.4 | 5.4 | 0.0 | 161.09 |
| Time center (s) | 176.6 | 7.7 | 47.5 | 299.3 | 124.1 | 9.7 | 37.6 | 250.7 |
| Time center + closed arms (s) | 248.0 | 5.9 | 136.0 | 299.3 | 180.5 | 10.4 | 65.6 | 295.0 |
| Time in immobility (s) | 224.8 | 5.4 | 127.3 | 299.25 | 175.3 | 7.7 | 88.13 | 263.2 |
| Average velocity open arms (m/s) | 0.033 | 0.003 | 0 | 0.100 | 0.055 | 0.003 | 0.019 | 0.098 |
| Average velocity closed arms (m/s) | 0.049 | 0.003 | 0 | 0.109 | 0.074 | 0.005 | 0 | 0.129 |
| Total distance covered (m) | 10.5 | 0.6 | 0.7 | 18.6 | 15.86 | 0.9 | 6.9 | 29.5 |
| Number of entries open arms (n) | 2.9 | 0.3 | 0 | 8 | 4.9 | 0.4 | 1 | 10 |
| Number of entries closed arms (n) | 2.8 | 0.2 | 0 | 7 | 3.0 | 0.2 | 0 | 7 |
