# Supplementary material for: An elevated plus-maze in mixed reality for studying human anxiety-related behavior
Source: BMC Biol. 2017 Dec 21;15:125. doi: 10.1186/s12915-017-0463-6 (PMC5740602; doi:10.1186/s12915-017-0463-6)
Supplement: Supplementary file 9 — Subjective ratings on a scale from 0 (not at all) to 9 (very strongly) that were collected after behavioral testing in Study 2. (DOCX 16 kb) [file 12915_2017_463_MOESM8_ESM.docx]

***Table S6.*** Subjective ratings on a scale from 0 = *not at all* to 9 = *very strongly* that were collected after behavioral testing in study 2*.*

| *Measures (0-9/9)* | ***Lorazepam*** | | ***Placebo*** | | **Yohimbine** | |
| --- | --- | --- | --- | --- | --- | --- |
|  | **Mean** | **SEM** | **Mean** | **SEM** | **Mean** | **SEM** |
| I immersed into the scene | 7.9 | 0.3 | 7.2 | 0.5 | 7.8 | 0.3 |
| I had anxiety | 3.3 | 0.5 | 4.9 | 0.6 | 5.7 | 0.7 |
| It was threatening | 4.1 | 0.6 | 5.6 | 0.5 | 5.9 | 0.6 |
| I moved precariously | 5.2 | 0.5 | 5.9 | 0.5 | 7.2 | 0.6 |
| I had panic | 0.9 | 0.2 | 1.8 | 0.6 | 2.4 | 0.6 |
| I felt inner tension | 3.5 | 0.6 | 5.1 | 0.4 | 4.8 | 0.7 |
| I have inner tension now | 1.2 | 0.4 | 1.4 | 0.3 | 2.8 | 0.5 |
| I had somatic symptoms (e.g. sweating) | 3.4 | 0.5 | 5.2 | 0.5 | 6.5 | 0.6 |
| I was paralyzed | 2.1 | 0.5 | 2.7 | 0.5 | 2.9 | 0.6 |
| I felt dizzy | 1.6 | 0.5 | 1.5 | 0.4 | 1.3 | 0.5 |
| I felt like being watched | 0.7 | 0.3 | 0.6 | 0.2 | 0.8 | 0.2 |
| I felt shame | 2.3 | 0.6 | 2.9 | 0.5 | 3.0 | 0.6 |
