# Supplementary material for: An elevated plus-maze in mixed reality for studying human anxiety-related behavior
Source: BMC Biol. 2017 Dec 21;15:125. doi: 10.1186/s12915-017-0463-6 (PMC5740602; doi:10.1186/s12915-017-0463-6)
Supplement: Supplementary file 10 — Side effects after medication in Study 2. Data were collected directly before behavioral testing (i.e., 55 min after medication). (DOCX 19 kb) [file 12915_2017_463_MOESM9_ESM.docx]

***Table S7.*** Side effects after medication in study 2. Data were collected directly before behavioral testing (i.e., 55min after medication).

|  | Treatment | Placebo | Lorazepam | Yohimbine |
| --- | --- | --- | --- | --- |
| *Side effect* | ***Severity*** | ***N (%)*** | ***N (%)*** | ***N (%)*** |
| Dry mouth | Missing  Not present  Very mild  Mild  Moderate  Strong  Very strong  Extreme | 1 (5.6)  13 (72.2)  2 (11.1)  1 (5.6)  0  0  0  0 | 0  13 (59.1)  2 (9.1)  6 (27.3)  1 (4.5)  0  0  0 | 1 (6.3)  12 (75.1)  2 (12.5)  1 (6.3)  0  0  0  0 |
| Dry Skin | Missing  Not present  Very mild  Mild  Moderate  Strong  Very strong  Extreme | 1 (5.6)  11 (61.1)  2 (11.1)  3 (16.7)  0  0  0  0 | 0  18 (81.8)  2 (9.1)  1 (4.5)  1 (4.5)  0  0  0 | 1 (6.3)  9 (56.3)  6 (37.5)  0  0  0  0  0 |
| Blurred vision | Missing  Not present  Very mild  Mild  Moderate  Strong  Very strong  Extreme | 1 (5.6)  14 (77.8)  1 (5.6)  1 (5.6)  0  0  0  0 | 0  21 (95.5)  0  1 (4.5)  0  0  0  0 | 1 (6.3)  13 (81.2)  1 (6.3)  0  1 (6.3)  0  0  0 |
| Sedation | Missing  Not present  Very mild  Mild  Moderate  Strong  Very strong  Extreme | 1 (5.6)  11 (61.1)  3 (16.7)  1 (5.6)  1 (5.6)  0  0  0 | 0  11 (50)  2 (9.1)  4 (18.2)  1 (4.5)  4 (18.2)*  0  0 | 1 (6.3)  11 (68.8)  2 (12.5)  1 (6.3)  1 (6.3)  0  0  0 |
| Nausea | Missing  Not present  Very mild  Mild  Moderate  Strong  Very strong  Extreme | 1 (5.6)  14 (77.8)  2 (11.1)  0  0  0  0  0 | 0  21 (95.4)  1 (4.5)  0  0  0  0  0 | 1 (6.3)  12 (75)  2 (12.5)  1 (6.3)  0  0  0  0 |
| Dizziness | Missing  Not present  Very mild  Mild  Moderate  Strong  Very strong  Extreme | 1 (5.6)  15 (83.3)  1 (5.6)  0  0  0  0  0 | 0  19 (86.4)  1 (4.5)  2 (9.1)  0  0  0  0 | 1 (6.3)  13 (81.3)  1 (6.3)  0  1 (6.3)  0  0  0 |
| Headache | Missing  Not present  Very mild  Mild  Moderate  Strong  Very strong  Extreme | 1 (5.6)  13 (72.2)  3 (16.7)  0  0  0  0  0 | 0  19 (86.4)  2 (9.1)  1 (4.5)  0  0  0  0 | 1 (6.3)  11 (68.8)  2 (12.5)  2 (12.5)  0  0  0  0 |
| Agitation | Missing  Not present  Very mild  Mild  Moderate  Strong  Very strong  Extreme | 1 (5.6)  10 (55.5)  3 (16.7)  2 (11.1)  1 (5.6)  0  0  0 | 0  16 (72.7)  3 (13.6)  2 (9.1)  1 (4.5)  0  0  0 | 1 (6.3)  14 (87.5)  0  1 (6.3)  0  0  0  0 |

*Participants indicating strong sedation before experimental testing were excluded
