# Supplementary material for: An elevated plus-maze in mixed reality for studying human anxiety-related behavior
Source: BMC Biol. 2017 Dec 21;15:125. doi: 10.1186/s12915-017-0463-6 (PMC5740602; doi:10.1186/s12915-017-0463-6)
Supplement: Supplementary file 11 — Results of questionnaire data from Study 2 Spielberger State-Trait Anxiety Inventory (STAI) trait subscore, acrophobia questionnaire (AQ), Liebowitz Social Anxiety Scale (LSAS), and Sensation Seeking Scale Form V (SSSV) including respective subscores, given in mean ± standard error. (DOCX 17 kb) [file 12915_2017_463_MOESM10_ESM.docx]

***Table S8.*** Results of questionnaire data from study 2 Spielberger’s state-trait-anxiety inventory trait subscore (STAI), acrophobia questionnaire (AQ), Liebowitz social anxiety scale (LSAS), Sensation Seeking Scale Form V (SSSV) including respective subscores, given in Mean ± SE: standard error.

| *Questionnaire* | ***All*** | | ***Lorazepam*** | | ***Placebo*** | | ***Yohimbine*** | |
| --- | --- | --- | --- | --- | --- | --- | --- | --- |
|  | **Mean** | **SEM** | **Mean** | **SEM** | **Mean** | **SEM** | **Mean** | **SEM** |
| STAI trait | 35.0 | 1.0 | 35.0 | 1.6 | 33.6 | 1.3 | 36.4 | 2.2 |
| AQ - avoidance | 3.5 | 0.9 | 2.3 | 0.6 | 2.9 | 1.1 | 5.6 | 2.4 |
| AQ - anxiety | 13.6 | 1.4 | 12.0 | 2.8 | 14.0 | 2.2 | 14.9 | 2.5 |
| AQ - total | 17.1 | 1.8 | 14.3 | 3.3 | 16.9 | 3.1 | 20.4 | 3.1 |
| SSSV - thrill and adventure seeking | 6.8 | 0.3 | 6.4 | 0.7 | 7.2 | 0.5 | 6.8 | 0.6 |
| SSSV - disinhibition | 5.8 | 0.3 | 5.8 | 0.6 | 5.8 | 0.6 | 5.9 | 0.4 |
| SSSV - experience seeking | 7.1 | 0.3 | 7.3 | 0.5 | 7.3 | 0.5 | 7.0 | 0.4 |
| SSSV - boredom susceptibility | 4.0 | 0.3 | 4.2 | 0.4 | 3.6 | 0.6 | 4.3 | 0.3 |
| SSSV - total | 23.7 | 0.9 | 23.7 | 1.6 | 23.6 | 2.0 | 23.9 | 1.0 |
| LSAS - anxiety | 14.5 | 1.5 | 13.1 | 2.4 | 17.5 | 2.8 | 12.8 | 2.3 |
| LSAS - avoidance | 14.9 | 1.3 | 13.8 | 2.4 | 17.3 | 2.1 | 13.4 | 2.6 |
| LSAS - total | 29.4 | 2.7 | 26.9 | 4.5 | 34.8 | 4.8 | 26.2 | 4.7 |
